# Identification of novel regulatory modules in dicotyledonous plants using expression data and comparative genomics

**DOI:** 10.1186/gb-2006-7-11-r103

**Published:** 2006-11-07

**Authors:** Klaas Vandepoele, Tineke Casneuf, Yves Van de Peer

**Affiliations:** 1 Department of Plant Systems Biology, Flanders Interuniversity Institute for Biotechnology (VIB), Ghent University, Technologiepark, B-9052 Ghent, Belgium

## Abstract

**Background:**

Transcriptional regulation plays an important role in the control of many biological processes. Transcription factor binding sites (TFBSs) are the functional elements that determine transcriptional activity and are organized into separable *cis*-regulatory modules, each defining the cooperation of several transcription factors required for a specific spatio-temporal expression pattern. Consequently, the discovery of novel TFBSs in promoter sequences is an important step to improve our understanding of gene regulation.

**Results:**

Here, we applied a detection strategy that combines features of classic motif overrepresentation approaches in co-regulated genes with general comparative footprinting principles for the identification of biologically relevant regulatory elements and modules in *Arabidopsis thaliana*, a model system for plant biology. In total, we identified 80 TFBSs and 139 regulatory modules, most of which are novel, and primarily consist of two or three regulatory elements that could be linked to different important biological processes, such as protein biosynthesis, cell cycle control, photosynthesis and embryonic development. Moreover, studying the physical properties of some specific regulatory modules revealed that *Arabidopsis *promoters have a compact nature, with cooperative TFBSs located in close proximity of each other.

**Conclusion:**

These results create a starting point to unravel regulatory networks in plants and to study the regulation of biological processes from a systems biology point of view.

## Abstract 2

A strategy combining classical motif overrepresentation in co-regulated genes with comparative footprinting is applied to identify 80 transcription factor binding sites and 139 regulatory modules in Arabidopsis thaliana.

## Background

Regulation of gene expression plays an important role in a variety of biological processes such as development and responses to environmental stimuli. In plants, transcriptional regulation is mediated by a large number (>1,500) of transcription factors (TFs) controlling the expression of tens or hundreds of target genes in various, sometimes intertwined, signal transduction cascades [1,2]. Transcription factor binding sites (TFBSs; or DNA sequence motifs, or motifs for short) are the functional elements that determine the timing and location of transcriptional activity. In plants and other higher eukaryotes, these elements are primarily located in the long non-coding sequences upstream of a gene, although functional elements in introns and untranslated regions have been described as well [3,4]. Moreover, regulatory motifs organize into separable *cis*-regulatory modules (CRMs; modules for sort), each defining the cooperation of several TFs required for a specific spatio-temporal expression pattern (for a review, see [5]). As a consequence of this complex organization, understanding the combinatorial nature of transcriptional regulation at a genomic scale is a major challenge, as the number of possible combinations between TFs and targets is enormous. On top of this, it is important to realize that not all motifs present in a promoter are functional elements or simultaneously active, since the cooperation between TFs is context dependent [6]. In the absence of already characterized TFBSs or systematic genome-wide location (that is, chromatin immunoprecipitation-chip) data revealing interactions between TFs and target genes, sequence and expression data are the only sources of information that can be combined to identify CRMs [7-9].

The discovery of regulatory motifs and their organization in promoter sequences is an important first step to improve our understanding of gene expression and regulation. Since co-expressed genes are likely to be regulated by the same TF, the identification of shared and thus overrepresented motifs in sets of potentially co-regulated genes provides a practical solution to discover new TFBSs. Complementarily, the identification of significantly conserved short sequences (or footprints) in the promoters of orthologous genes in related species points to candidate regulatory motifs for a particular gene [10]. In yeasts and animals both overrepresentation of motifs in co-regulated genes and comparison of orthologous sequences have been successfully applied to delineate regulatory elements (for an overview, see [11,12]); in plants, however, mainly analyses on co-regulated genes for particular biological processes (for example, stress, hormone and light-response, cell cycle control) have been reported [2].

Two problems interfering with comparative approaches for the detection of regulatory motifs in orthologous plant sequences are the limited amount of genomic sequence information for related species (but see [13]) and the high frequency of both small- and large-scale duplication events that hamper the delineation of correct orthologous relationships [14,15]. Finally, the correct identification of functional TFBS is more complex in higher eukaryotes compared to prokaryotes or yeast because of the longer intergenic sequences. Consequently, characterizing properties of regulatory elements and modules is not trivial due to the inclusion of large amounts of false positives in sets of putative target genes. To overcome these problems, several approaches integrate local sequence conservation between orthologous upstream regions to exclude non-conserved regions from the search space and to make more accurate predictions about the presence of regulatory signals [16-21]. Nevertheless, this methodology requires that genomic data from closely related species are available and that correct (one-to-one) orthologous relationships can be identified for nearly all genes.

Here, we present a detection strategy that integrates features of classic approaches looking for overrepresented motifs with general comparative footprinting principles for the systematic characterization of biologically relevant TFBSs and CRMs in *Arabidopsis thaliana*, a dicotyledonous plant model system. In a first stage, a classic Gibbs-sampling approach is used to identify TFBSs in sets of co-expressed genes. Next, these TFBSs are presented to an evolutionary filter to select functional regulatory elements based on the global conservation of TFBSs in target genes in a related species, *Populus trichocarpa *(poplar). In a second stage, a two-way clustering procedure combining the presence/absence of motifs and expression data is used to identify additional new TFBSs. The Gene Ontology (GO) vocabulary combined with the original expression data is used to functionally annotate sets of genes containing a particular regulatory element or module. As a result, 80 TFBSs are reported, of which more than half correspond with previously described plant *cis*-regulatory elements. More interesting, we were able to identify numerous regulatory modules driving different biological processes, such as protein biosynthesis, cell cycle, photosynthesis and embryonic development. Finally, the physical properties of some modules are characterized in more detail.

## Results and discussion

### General overview

**Figure 1:**
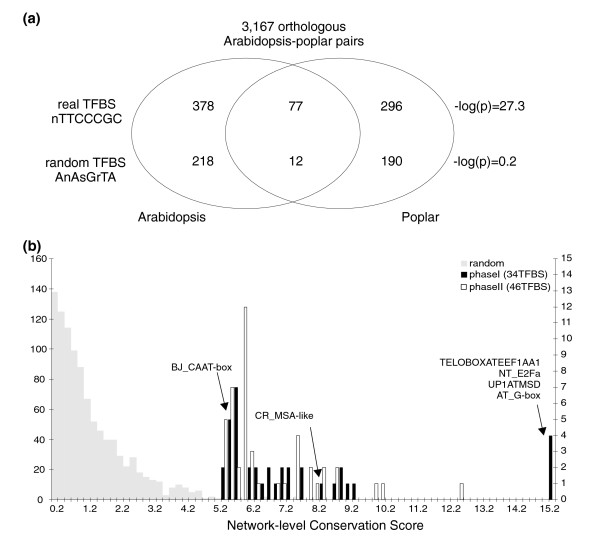
Network-level conservation filter. **(a) **The occurrence of a candidate TFBS in the set of orthologous *Arabidopsis*-poplar gene pairs was determined and the significance of the overlap is measured using the hypergeometric distribution [24]. The NCS is defined as the negative logarithm of the hypergeometric *p *value. **(b) **Distribution of NCS values for 1,000 randomly generated TFBSs (grey) and the motifs found using the co-expression (black) and the two-way clustering (white) procedure. The left and right y-axis show the frequency for the random and the potentially functional TFBSs, respectively.

The input data for our analysis were genome-wide expression data and the genome sequence from *Arabidopsis*, plus genomic sequence data from a related dicotyledon, poplar [22]. Whereas the expression data are required for creating sets of co-regulated genes that serve as input for the detection of TFBSs using MotifSampler (see Materials and methods), the genomic sequences are used to delineate orthologous gene pairs between *Arabidopsis *and poplar, forming the basis for the evolutionary conservation filter. This filter is used to discriminate between potentially functional and false motifs and is based on the network-level conservation principle, which applies a systems-level constraint to identify functional TFBSs [23,24]. Briefly, this method exploits the well-established notion that each TF regulates the expression of many genes in the genome, and that the conservation of global gene expression between two related species requires that most of these targets maintain their regulation. In practice, this assumption is tested for each candidate motif by determining its presence in the upstream regions of two related species and by calculating the significance of conservation over orthologous genes (see Materials and methods; Figure 1a). Whereas the same principle of evolutionary conservation is also applied in phylogenetic footprinting methods to identify TFBSs, it is important to note that, here, the conservation of several targets in the regulatory network is evaluated simultaneously. This is in contrast with standard footprinting approaches, which only use sequence conservation in upstream regions on a gene-by-gene basis to detect functional DNA motifs.

**Figure 2:**
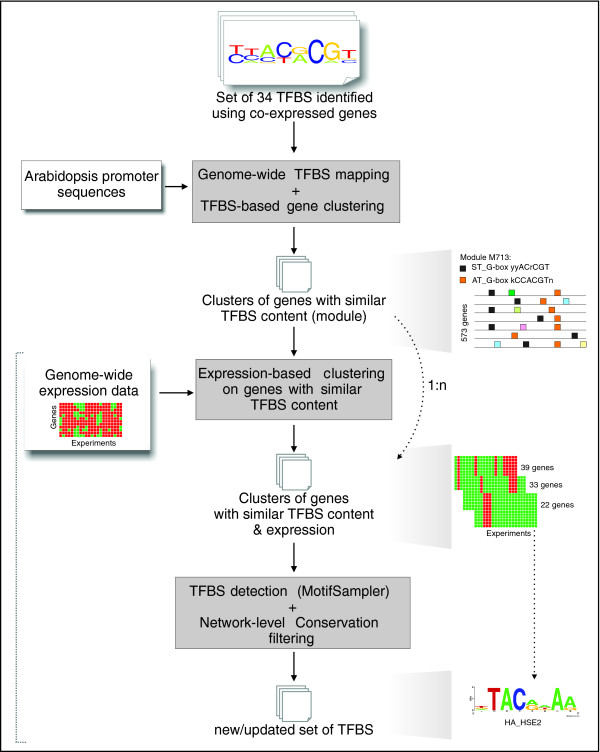
Detection of TFBSs using two-way clustering. Starting from the available set of 34 TFBSs identified using sets of co-expressed genes (see text for details), clusters of genes with similar TFBS combinations in their promoter are delineated. Next, within each set of genes with similar TFBS content, groups of co-expressed genes are identified. Finally, motif detection is applied and evolutionarily conserved TFBSs are retained. The panel on the right shows the identification of the TFBS HA_HSE2 involved in zygotic embryogenesis. The top picture depicts a subset of all 573 *Arabidopsis *genes containing the module consisting of two distinct G-boxes. The two images below show the three groups of co-expressed genes and the newly identified TFBSs found in a set of 22 genes containing both G-boxes in their promoter and showing embryo-specific expression. Note that the section indicated with the dotted line corresponds with the motif-detection approach applied on co-expressed genes in the first stage.

After applying motif detection on a set of co-expressed *Arabidopsis *genes in a first stage, all TFBSs retained by the network-level conservation filter are subsequently combined with the original expression data to identify CRMs and additional regulatory elements ('two-way clustering'; Figure 2). Both objectives were combined because it has been demonstrated that the task of module discovery and motif estimation is tightly coupled [25]. We reasoned that, for a group of genes with similar motif content but with dissimilar expression profiles, additional TFBSs may exist that explain the apparent discrepancy between motif content and expression profile.

Whereas the procedure for detecting TFBS in co-expressed genes combined with the evolutionary filter is highly similar to the methodology described by Pritsker and co-workers [23], the second stage of TFBS detection using the two-way clustering procedure is, to our knowledge, novel. The inference of regulatory modules is related to the work of Kreiman [18], although, in the current study, no *a priori *physical constraints were used to exhaustively search for CRMs.

### Identification of individual TFBSs using co-expressed genes

Applying the Cluster Affinity Search Technique (CAST) algorithm to the data set measuring the expression of 19,173 *Arabidopsis *genes over 489 different experiments (1,168 Affymetrix ATH1 slides; see Additional data file 5) yielded 122 clusters of co-regulated genes covering 5,664 genes (see Materials and methods). After running MotifSampler, applying the network-level conservation filter and removing redundant motifs (see Materials and methods), 34 motifs with a significant (*p *value < 0.01) Network-level Conservation score (NCS) were retained (Figure 1b). Interestingly, 25 of the identified TFBSs can be functionally annotated based on overrepresented GO Biological Process or Molecular Function terms in the set of putative target genes (Table 1). Overall, nearly 60% (20/34) of all motifs correspond with known plant regulatory elements. Throughout this paper, for motifs corresponding with known regulatory elements described in PLACE [26] and PlantCARE [27] the original name is used, whereas for new elements the consensus motif will be used.

The telo-box (TELOBOXATEEF1AA1) is the TFBS with the highest NCS value (40.06), indicating that this motif is highly conserved in orthologous target genes between *Arabidopsis *and poplar. The GO annotation reveals that this motif is highly enriched in the promoter of genes involved in ribosome biogenesis and assembly (*p *value < 10^-12^; 4.4-fold enrichment), confirming the role of the telo-box in regulating components of the translational machinery [28]. Other motifs with high NCS values together with their functional annotation correspond to well-described plant TFBSs, such as the E2F box and the MSA element involved in DNA replication and microtubule motor activity during the cell cycle [29], the UP1 box mediating the transcription of protein synthesis [30], and the G box inducing the transcription of photosynthesis genes in response to light [31]. The observation that 71% of these motifs are located within the first 500 base-pairs (bp) upstream of the translation start site (Additional data file 1) for conserved orthologous *Arabidopsis*-poplar targets confirms previous findings that *Arabidopsis *promoters are generally compact [32,33].

### Combining motif and expression data to identify additional TFBSs

Although the motif detection approach using co-expressed genes revealed a first set of TFBSs, it is clear that expression data alone are insufficient to unravel the complex nature of transcriptional regulation in higher plants. Therefore, we applied a two-way clustering procedure combining motif and expression data to identify additional regulatory elements. We again used MotifSampler combined with the network-level conservation filter to identify potential TFBSs in clusters of co-expressed genes, but now also incorporated the prior knowledge about the presence of particular TFBSs in a gene's promoter. Thus, first all genes with a particular motif combination (module) in the *Arabidopsis *genome were identified after which the expression profiles of these genes were used to delineate subgroups of co-expressed genes, which were then again presented to the motif detection routine (MotifSampler and network-level conservation filter; Figure 2). The rationale behind this approach is that additional TFBSs may exist that explain the different expression patterns within the set of genes containing the same module. As shown below, these new motifs can be missed in the first detection stage on co-expressed genes since the fraction of genes containing this TFBS within the set of co-expressed genes is too small for reliable detection by MotifSampler. By evaluating all possible combinations (from two up to four motifs) using all 34 initial TFBSs, we found 1,249 modules containing more than 40 genes. Next, we determined groups of co-expressed genes for each set of genes characterized by a specific module using the CAST algorithm (as described before). In total, 695 regulons, containing genes with a particular module and similar expression profiles, were found, covering 4,100 *Arabidopsis *genes. Note that the way of grouping genes with identical modules is compatible with the combinatorial nature of transcriptional control in higher eukaryotes, since the presence of additional TFBSs in a gene's promoter does not interfere with the gene clustering based on TFBS content (for example, gene *i *with motifs A, B and C can theoretically occur in the clusters containing module A-B, A-C, B-C and A-B-C; see Materials and methods).

After running MotifSampler and the network-level conservation filter on all regulons, 46 new TFBSs were found (Additional data file 6). Again, the high fraction (25/46, or 54%) of TFBSs with similarity to previously described ones indicates that we most probably identified an extra set of genuine regulatory elements. As an illustration, we discuss the discovery of the HA_HSE2 motif, which is an element inducing gene expression during zygotic embryogenesis [34]. Initially, 573 *Arabidopsis *genes were grouped containing a combination of two distinct G-boxes in their promoters (AT_G-box kCCACGTn and ST_G-box yyACrCGT; Table 1). Subsequent clustering of the expression profiles of these genes, enriched for the GO terms embryonic development (sensu Magnoliophyta) and seed development (both with *p *value < 10^-2^; 7.4-fold and 8.1-fold enrichment, respectively), yielded three regulons, of which one showed expression in seeds, a second one expression in leaves and shoots, and a third one expression in the globular and heart stage embryo. Running the motif detection routine on the 22 genes in this last regulon resulted in the discovery of the HA_HSE2 motif (NCS 7.91). This motif was not identified in the first TFBS detection run using expression data only, since the genes in this regulon were part of a big set of 645 co-expressed genes not yielding any significant TFBSs. This finding confirms that splitting up co-expressed genes into smaller subsets based on prior knowledge of motif content can enhance the identification of new TFBSs.

### Inferring functional regulatory modules

**Figure 3:**
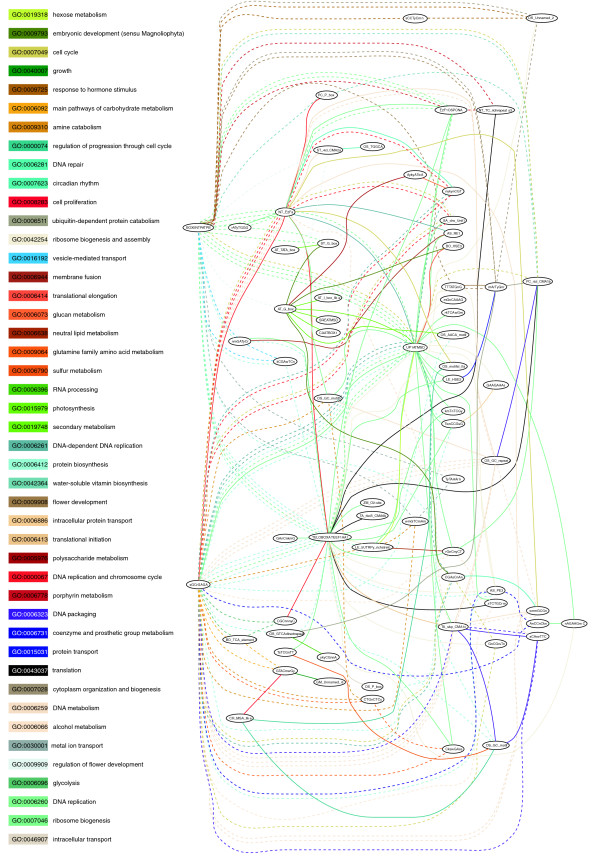
Motif synergy map for 139 modules with significant GO Biological Process annotation. The full and dotted lines connect motifs cooperating in modules containing two and three TFBSs, respectively. Line colors indicate the GO Biological Process enrichment for *Arabidopsis *genes containing this module (see also Additional data file 7).

To get a general overview of the involvement of all 80 TFBSs (34 from co-expressed genes in the first stage plus 46 from two-way clustering in the second stage) and the derived CRMs in different biological processes, we identified all modules with two to four motifs (containing at least 20 *Arabidopsis *genes) and again used overrepresented GO terms for functional annotation. Briefly, we selected all *Arabidopsis *genes with a particular motif combination present in their upstream regions and verified whether any GO Biological Process term was significantly enriched within this set of putative target genes. Figure 3 shows the motif synergy map depicting the cooperation of different TFBSs for which the GO enrichment score is stronger for the module than for the individual TFBS (within that module). Applying this criterion is necessary to specifically identify the functional properties of the module, because the GO enrichment for many modules is caused by the presence of an individual TFBS and not by the specific TFBS combination in the CRM. In total, 139 modules with significant functional GO Biological Process enrichment were identified, of which 97 consist of a combination of two and 42 of three TFBSs (Additional data file 7). Moreover, 69 identified TFBSs in this study could be allocated to one or more CRM with significant functional annotation. The module with the strongest GO enrichment in the synergy map consists of a telo-box and the UP1 motif and targets protein biosynthesis (*p *value < 10^-51^) and ribosome biogenesis (*p *value < 10^-25^) genes (for example, 40S and 60S ribosomal proteins, translation initiator factors). In total, 851 *Arabidopsis *genes contain this module and the expression coherence [9] of these genes (EC = 0.14; see Materials and methods) illustrates that this module is responsible for similar expression profiles in a large number of these genes. Detailed information about target genes and functional annotation for the different CRMs can be consulted on our website [35].

**Figure 4:**
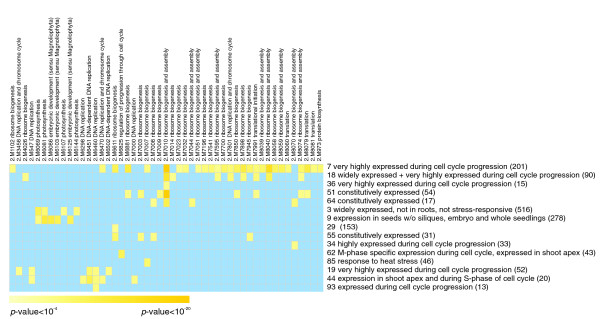
Correlation between *cis*-regulatory modules and clusters of co-expressed genes. Rows depict co-expression clusters with their corresponding cluster number and brief description, if available, whereas columns show modules with their corresponding GO descriptions. The number of genes within each co-expression cluster is indicated in parentheses. Only expression clusters enriched for one (or more) modules are shown. Enrichment was calculated using the hypergeometric distribution and *p *values were corrected for multiple hypotheses testing with the false discovery rate method (q-value) [76].

Analyzing the topology of the motif synergy map reveals some highly connected TFBSs (for example, UP1ATMSD, TELOBOXATEEF1AA1, sGCrGAGA, BOXIINTPATPB, AT_G-box kCCACGTn), which control, in cooperation with other TFBSs, different biological processes. A set of modules contain a G-box and confirm its role in controlling light-dependent processes such as photosynthesis (module 2.M6107, AT_G-box kCCACGTn + I-box-like ATAATCCA; module 2.M6144, AT_G-box kCCACGTn + OS_AACA_motif; module 2.M6069, AT_G-box kCCACGTn + SREATMSD) and embryonic development (module 2.M6103, AT_G-box kCCACGTn + CGAsCnAn; module 2.M6125, AT_G-box kCCACGTn + BO_HSE3 box). The cooperation between the G-box and the I-box-like motif in the module with GO enrichment 'photosynthesis' targets genes coding for chlorophyll binding proteins, different photosystem I reaction center subunits, photosystem II associated proteins, and ferredoxin. The high expression of these genes in plant tissues exposed to light suggests a function for this module as a composite light-responsive unit [36]. Combining the clusters of co-expressed genes used in the first detection stage with the targets of the different modules (Figure 4) shows a highly significant overlap of expression cluster 3 with the photosynthesis modules 2.M6069, 2.M6144, 2.M6107 and 2.M6081 (AT_G-box kCCACGTn + UP1 box). These strong associations indicate that these motif combinations are involved in (light-regulated) primary energy production.

Three modules (2.M6086, 2.M6103 and 2.M6125) targeting genes involved in embryonic development (>7-fold GO enrichment; Additional data file 7) are strongly associated with expression cluster 9, which shows high transcriptional activity in seedlings and embryo (Figure 4). The presence of these modules, all containing a G-box, in some well-described embryogenesis genes within this expression cluster (for example, late embryogenesis-abundant proteins, zinc-finger protein PEI1 and NAM transcriptional regulators [37,38]) confirms our finding that these modules play an important role in transcriptional control during embryo development.

The motif sGCrGAGA is involved in 26 different modules and is, to our knowledge, a new TFBS. Whereas the full set of *Arabidopsis *genes containing this motif shows a functional enrichment for 'energy derivation by oxidation of organic compounds' (Table 1), more than a quarter of all modules (7/26) containing this regulatory element seem to have a role in transcriptional control of sugar, amino acid or alcohol metabolism. Examples of biosynthesis pathways mediated by these modules according to the GO Biological Process annotation include glycolysis, amine catabolism and branched chain family amino acid metabolism (Additional data file 7).

Another module (2.M6825) controls the progression through the cell cycle and consists of a combination of the known MSA element together with the OS_GC motif. A large number of genes associated with mitosis and cytokinesis, such as those encoding B-type cyclins, kinesin motor proteins and microtubule and phragmoplast-associated proteins, contain this CRM and are linked with expression cluster 62 (Figure 4). Comparing the occurrence of this module in a set of approximately 1,000 periodically expressed genes determined in *Arabidopsis *cell suspensions by Menges and co-workers [39] confirms a strong enrichment towards M-phase specific genes (hypergeometric probability distribution; *p *value < 10^-21^). Nevertheless, because the frequency of the individual MSA element is higher in the set of M-phase specific genes compared to the occurrence of the module (87/198 MSA element and 40/198 module, respectively), this indicates that the presence of the individual MSA box is sufficient for M-phase expression during cell division and that additional cooperative elements only moderately mediate the level of transcription, as recently shown [40]. Likewise, despite the fact that several modules (for example, 2.M547, 2.M6460 and 2.M6451) consisting of the NT_E2Fa motif and one or more cooperative TFBS are targeting genes involved in DNA replication (>10-fold enrichment) and are strongly associated with expression cluster 44 (Figure 4) containing many DNA replication genes (for example, DNA replication licensing factor, PCNA1-2), it is currently unclear whether additional motifs, apart from one or more E2F elements, are essential for transcriptional induction during S-phase in plants [33].

Another module driving endogenous light-regulated response contains the ST_4cl-CMA2a and OS_TGGCA boxes and targets genes involved in circadian rhythm (2.M8255, 'circadian rhythm' >24-fold enrichment). Examples of genes containing this module are CONSTANS, a zinc finger protein linking day length and flowering [41], as well as APRR5 and APRR7, pseudo-response regulators subjected to a circadian rhythm at the transcriptional level [42]. One of the TFBSs within this module, motif OS_TGGCA with sequence [GT]C [AT]A [AG]TGG, is highly similar to the SORLIP3 motif (CTCAAGTGA; Pearson correlation coefficient (PCC) = 0.56 between linearized PWM and SORPLIP3), a sequence found to be overrepresented in light-induced promoters [43].

### Properties of cis-regulatory modules

Due to the frequent nature of large-scale duplication events in plants, a one-to-one orthologous relationship with poplar could be ensured for only a minority of *Arabidopsis *genes (17%). Therefore, applying across-species conservation on a genome-wide scale to predict functional TFBSs, as done in mammals and yeast, is not straightforward in plants. Similarly, studying cooperative TFBSs within regulatory modules also suffers from the inclusion of potentially false-positives when selecting genes in one species containing a putative module. Therefore, we exploited the conservation of TFBSs between *Arabidopsis *and poplar orthologs to study the properties of some modules in more detail. Based on all 139 modules and the set of 3,167 (one-to-one) orthologous genes between *Arabidopsis *and poplar, we only retained 30 modules with five or more conserved target genes for further analysis. By applying this stringent filtering step of five or more conserved orthologous targets, we wanted to study the physical properties - motif order and spacing - of CRM in a set of *Arabidopsis *target genes enriched for functional TFBSs (and with a minimum number of false-positives; data not shown). Since no *a priori *information about such properties was included in the identification of TFBSs and CRMs, we used this data set to verify whether such constraints exist and are used by the transcriptional apparatus to control gene expression in plants.

**Figure 5:**
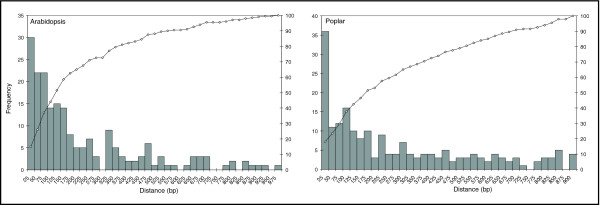
Motif distance distributions for 30 conserved modules in orthologous target genes between *Arabidopsis* and poplar. For all modules, the distance (in bp) between cooperative TFBS was measured in 200 conserved orthologous target genes and plotted in a histogram for Arabidopsis and poplar. The white boxes denote the cumulative fraction.

First, for each module the overrepresented motif order was quantified in all conserved target genes (for example, 9/11 of all conserved *Arabidopsis *target genes for module 2.M7010 contain pattern [TELOBOXATEEF1AA1 *spacer *UP1ATMSD *spacer *start codon]). Grouping all these results indicates that, on average, 68% (136/200) of all *Arabidopsis *targets contain an overrepresented motif order (Additional data file 8). Nevertheless, the observation that, on average, approximately 64% of the orthologous poplar targets contain the same motif order suggests that, although a preferred motif order might be present for some modules (Additional data file 2), this configuration is evolutionarily rather weakly conserved. Measuring the distance between cooperative TFBSs reveals that, for 11/30 modules, the average distance is significantly smaller than expected by chance (Additional data file 8). Moreover, the overall distribution of distances between TFBSs measured for all 200 targets within these 30 modules is, in both *Arabidopsis *and poplar, significantly different from a random distribution (Mann-Whitney *U *test *p *value < 0.001; Figure 5). This indicates that, like in other eukaryotic species (for example, [18,44,45]), the distance between cooperative motifs within a module is important for functionality.

## Conclusion

The results of this study confirm that TFBS detection using expression data within an evolutionary context offers a powerful approach to study transcriptional control [18,20,23]. Especially, the exploitation of sequence conservation between related species offers a good control against false-positives when performing motif detection on co-regulated genes [46-49]. Using clusters of co-expressed genes, MotifSampler, two-way clustering and the network-level conservation principle, 80 distinct TFBSs could be identified, of which 45 correspond to known plant *cis*-regulatory elements. From these, 139 regulatory modules with biological functional annotation could be inferred and several CRMs were highly associated with distinct expression patterns. Despite the limited amount of comparative sequence data for dicotyledonous plants, which hinders the systematic identification of conserved and probably functional binding sites within a promoter, the regulatory modules identified here suggest that, like in yeast and animals, combinatorial transcriptional control plays an important role in regulating transcriptional activity in plants. For sure, the application of more advanced CRM detection methods (for example, [25,50,51]) integrating physical constraints acting on CRMs (as shown here) on more detailed expression data will lead to the discovery of additional plant CRMs. Finally, the sequencing of additional and less diverged plant species in the near future [52] should provide a more solid comparative framework to study the organization and evolution of transcriptional regulation within the green plant lineage.

## Materials and methods

### Expression data

A total of 1,168 Affymetrix ATH1 microarrays monitoring the transcriptional activity of more than 22,000 *Arabidopsis *genes in different tissues and under different experimental conditions were retrieved from the Nottingham *Arabidopsis *Stock Centre (NASC [53]; 1,151 slides) and The *Arabidopsis *Information Resource (TAIR [54]; 17 slides). An overview of all data sets is shown in Additional data file 5. Raw data were normalized using the MicroArray Suite 5.0 (MAS) implementation in Bioconductor ('mas5' function) [55]. To remove potentially cross-hybridizing probes, only genes for which a unique probe set is available on the ATH1 microarray (probe sets with a '_at' extension without suffix) were retained. Next, the genes were filtered based on the detection call that is assigned to each gene by the 'mas5calls' function implemented in Bioconductor. This software evaluates the abundance of each transcript and generates a detection *p *value indicating whether a transcript is reliably detected (*p *value < 0.04 for present value). Only genes that were called present in at least 2% of the experiments were retained for further analysis. Finally, the mean intensity value was calculated for the replicated slides, resulting in 489 measurements for 19,173 genes in total.

### Clustering of expression data

To group genes with similar expression profiles, we used the CAST algorithm with the PCC as affinity measure [56]. Advantages of CAST clustering over more classic algorithms such as hierarchical or K-means clustering are that only two parameters have to be specified (the affinity measure, here defined as PCC ≥ 0.8, and the minimal number of genes within a cluster, here set to 10) and that it independently determines the total number of clusters and whether a gene belongs to a cluster. We used an additional heuristic to choose the gene with the maximum number of neighbors (that is, the total number of genes having a similar expression profile) to initiate a new cluster. An overview of the cluster stability when randomly removing experiments from the complete expression data set is given in Additional data file 3.

### Detection of transcription factor binding sites

For each cluster S, grouping n_S _co-regulated genes returned by the CAST algorithm, we used MotifSampler [57] to identify an initial set of TFBSs. We restricted the search to the first 1,000 bp upstream of the translation start site. For some genes the upstream sequence was shorter because the adjacent upstream gene is located within a distance smaller than 1,000 bp. The parameters used were 6th order background model (computed from all *Arabidopsis *upstream sequences), -n 2 (number of different motifs to search for), -r 100 (number of times the MotifSampler should be repeated) and -w (length of the motif) set to 8nt. For each cluster, the 20 best and non-redundant motifs (represented as a position weight matrix (PWM)) according to their log-likelihood score were retained using MotifRanking (default parameters; shift parameter -s set to 2).

To create a non-redundant set of all motifs found in the different clusters of co-expressed genes, we first compared the similarity between two motifs as the PCC of their corresponding PWM. Each motif of length w was represented using a single vector, by concatenating the rows of its matrix (obtaining a vector of length 4*w). Subsequently, the PCC between every alignment of two motifs was calculated, as they are scanned past each other, in both strands [18,58]. Then, all motifs with a PCC >0.75 were considered as similar and only the motif with the highest NCS (see below) was retained.

The presence of a motif (represented by its corresponding PWM) in a DNA sequence was determined using MotifScanner, which uses a probabilistic sequence model (default parameters; prior probability -p set to 0.1). Both MotifRanking and MotifScanner, together with MotifSampler, are part of the INCLUSIVE package [59].

### Clustering based on TFBS content

To group genes containing similar motifs in their promoter and incorporating the possibility that not all motifs in a promoter are functional, we generated all groups of genes having two or more motifs in common. Starting from the set of non-redundant motifs mapped on all promoters, all motif combinations from two to four motifs were generated and only clusters with at least 20 genes containing that combination were retained. Note that, for a particular motif combination, the presence of additional motifs in a gene's promoter was ignored, resulting in the creation of overlapping clusters.

### Network-level conservation score

We identified 3,167 orthologous *Arabidopsis*-poplar gene pairs through phylogenetic tree construction (see below). Due to the high frequency of gene duplication in both *Arabidopsis *and poplar [60-62], we preferred to apply phylogenetic tree construction to delineate orthologous relationships instead of sequence similarity approaches based on reciprocal best hit (for example, [24,63]). Whereas the latter only uses similarity or identity scores to define putative orthology and is highly sensitive to incomplete associations due to in-paralogs, tree construction methods use an evolutionary model to estimate evolutionary distances and give a significance estimate through bootstrap sampling.

For each candidate TFBS and for all *Arabidopsis*-poplar orthologs, we first identified the set of *Arabidopsis *genes that have at least one occurrence matching the PWM in their upstream regions. Then, we also identified the poplar genes that have at least one occurrence matching the PWM in their upstream regions. Next, we calculated the overlap of matches in orthologs between both sets of sequences. Note that the matches can be anywhere in the upstream region and on any strand. For both *Arabidopsis *and poplar, the search was again restricted to the first 1,000 bp upstream from the translation start site or to a shorter region if the adjacent upstream gene is located within a distance smaller than 1,000 bp. The statistical significance of the overlap, which will be high for PWM representing functional TFBSs according to the network-level conservation principle, is measured using the hypergeometric distribution (for details, see [24]). Because the NCS, which is defined as the negative logarithm of the hypergeometric *p *value, is a relative measure of network-level conservation, the observed scores are compared against a distribution of scores obtained from random motifs. Thousand random motifs were generated by running the MotifSampler on clusters containing randomly selected genes. All NCS values larger than 5.3, which correspond to the 99th percentile of the random NCS distribution, were considered as significant.

### Orthology determination

The full proteomes (that is, all proteins in a genome) of *Arabidopsis*, poplar, rice, and *Ostreococcus tauri*, together with proteins inferred from cDNA sequences for *Pinus taeda*, *Pinus pinaster *and *Physcomitrella patens *were used to delineate gene families using protein clustering. First, an all-against-all sequence comparison was performed using BLASTP [64] and relevant hits were retained [65]. Briefly, two proteins are considered homologous only when they share a substantially conserved region on both molecules with a minimum amount of sequence identity. In this manner, multi-domain proteins for which the sequence only partially overlaps because of shared single protein domains, which occasionally leads to significant E-values in BLAST searches, are not retained as homologs. The proportion of identical amino acids in the aligned region between the query and target sequence is recalculated to I' = I × Min(n_1_/L_1_, n_2_/L_2_), where L_i _is the length of sequence i and n_i _is the number of amino acids in the aligned region of sequence i. This value I' is then used in the empirical formula for protein clustering proposed by Rost [66]. Finally, all valid homologous protein pairs are subject to a simple-linkage clustering routine to delineate protein gene families. *Arabidopsis *and rice sequences were downloaded from TIGR (releases 5.0 and 3.0, respectively), *Ostreococcus *sequences from [67,68], poplar sequences from the JGI consortium [69], and pine and moss data from the Sequence platform for Phylogenetic analysis of Plant Genes database (SPPG) [70]. The coding sequences for *Ostreococcus *and poplar correspond to the genes predicted by the EuGene gene prediction software [71].

For all 7,038 gene families containing one or more *Arabidopsis *and poplar gene (and covering in total 20,273 and 31,894 genes, respectively), protein multiple alignments were created using T-coffee [72]. Alignment columns containing gaps were removed when a gap was present in >10% of the sequences. To reduce the chance of including misaligned amino acids, all positions in the alignment left or right of the gap were also removed until a column in the sequence alignment was found where the residues were conserved in all genes included in our analyses. This was determined as follows: for every pair of residues in the column, the BLOSUM62 value was retrieved. Next, the median value for all these values was calculated. If this median was ≥0, the column was considered as containing homologous amino acids. Neighbor-Joining phylogenetic trees were constructed with PHYLIP [73] using the Dayhoff PAM matrix and 100 bootstrap samples. Trees were rooted if a non-dicotyledonous species was present within the gene family. In total, 3,167 orthologous gene pairs were identified as speciation nodes in the trees grouping one *Arabidopsis *and one poplar gene with high bootstrap support (≥70). An overview of the one-to-many and many-to-many orthologous relationships is shown in Additional data file 4. Note that these 3,167 orthologous gene pairs are not biased towards a particular functional GO class and thus can be used to estimate the conservation of candidate TFBSs between both plant genomes.

### Functional annotation

GO [74] associations for *Arabidopsis *proteins were retrieved from TIGR [75]. The assignments of genes to the original GO categories were extended to include parental terms (that is, a gene assigned to a given category was automatically assigned to all the parent categories as well). All GO categories containing less than 20 genes were discarded from further analysis. Enrichment values were calculated as the ratio of the relative occurrence in a set of genes to the relative occurrence in the genome. The statistical significance of the functional enrichment within sets of genes was evaluated using the hypergeometric distribution adjusted by the Bonferroni correction for multiple hypotheses testing. Corrected *p *values smaller than 0.05 were considered significant. Only CRMs with significant GO Biological Process annotation and an enrichment score higher than 5 were retained in the final data set.

### Expression coherence

The expression coherence, which is a measure of the amount of expression similarity within a set of genes, was calculated as described by Pilpel and co-workers [9]. Here, the PCC was used as a measure for similarity between expression profiles instead of the Euclidian distance used in the original implementation. Based on the similarity between expression profiles for 1,000 random genes (1,000 × 999 × 0.5 gene pairs), a PCC threshold of 0.5 (corresponding with the 95th percentile of this random distribution) was used to detect significantly co-expressed genes.

## Additional data files

The following additional data are available with the online version of this paper. Additional data file 1 is a figure showing the location of 34 conserved motifs (found in co-expressed genes) in *Arabidopsis *promoters (2,445 genes) and of all conserved motifs in *Arabidopsis *promoters with more than 3 kb un-annotated upstream space (with distance <1,000 bp between position in *Arabidopsis *and poplar; 125 genes). Additional data file 2 is a figure giving an overview of the motif organization in orthologous *Arabidopsis *(left) and poplar (right) targets for module 2.M7010. Additional data file 3 is a figure showing the stability of clusters of co-expressed genes when randomly removing experiments from the complete expression data set. Additional data file 4 is a figure that gives an overview of the number of one-to-many and many-to-many orthologous relationships in the phylogenetic trees. Additional data file 5 is a table giving an overview of the 489 *Arabidopsis *microarray experiments. Additional data file 6 is a table giving an overview of the TFBSs identified using two-way clustering. Additional data file 7 is a table giving an overview of the 139 *cis*-regulatory modules. Additional data file 8 is a table showing the motif order and spacing for 30 *cis*-regulatory modules.

## Supplementary Material

Additional data file 1 **(a) **Location of 34 conserved motifs (found in co-expressed genes) in *Arabidopsis *promoters (2,445 genes). **(b) **Location of all conserved motifs in *Arabidopsis *promoters with more 3 kb un-annotated upstream space (with distance <1,000 bp between position in *Arabidopsis *and poplar; 125 genes) Click here for file

Additional data file 2 Overview of motif organization in orthologous *Arabidopsis *(left) and poplar (right) targets for module 2.M7010 Click here for file

Additional data file 3 Stability of clusters of co-expressed genes when randomly removing experiments from the complete expression data set Click here for file

Additional data file 4 Overview of the number of one-to-many and many-to-many orthologous relationships in phylogenetic trees Click here for file

Additional data file 5 Overview of the 489 *Arabidopsis *microarray experiments Click here for file

Additional data file 6 Overview of the TFBS identified using two-way clustering Click here for file

Additional data file 7 Overview of the 139 cis-regulatory modules Click here for file

Additional data file 8 The white boxes denote the cumulative fraction Click here for file

## Figures and Tables

**Table 1:** Overview of the TFBSs identified using co-expressed genes

TFBS motif*	NCS^†^	Known motif	Site^‡^	Functional enrichment targets: GO Biological Process or Molecular Function^§^
nrCAAnTC (a)	5.77	BJ_CAAT-box	**TGCAAATC**T	GO:0008152 metabolism 8.58E-04 (1.2); GO:0003824 catalytic activity 8.91E-05 (1.2)
GTACAwry (b)	5.64			GO:0007275 development 2.89E-02 (1.6); GO:0003824 catalytic activity 2.98E-03 (1.2)
TTCkwwTs	5.79	BOXIINTPATPB	**ATAGAA**	
sGCrGAGA	5.77			GO:0015980 energy derivation by oxidation of organic compounds 4.82E-02 (2.7); GO:0008152 metabolism 1.43E-03 (1.2); GO:0003824 catalytic activity 2.89E-03 (1.1)
kCCACGTn (4)	17.54	AT_G-box; HV_ABRE6; PH_boxII	**GCCACGTG**GA; **GCCACGTA**CA; **TCCACGTG**GC	GO:0015979 photosynthesis 2.48E-04 (4.2); GO:0048316 seed development 2.64E-03 (3.6); GO:0009793 embryonic development (sensu Magnoliophyta) 6.15E-03 (3.5)
yCATTTnT (c)	8.7	GM_Unnamed_6	G**CATTTTT**ATCA	GO:0003700 transcription factor activity 2.94E-03 (1.3); GO:0030528 transcription regulator activity 1.64E-02 (1.3); GO:0003677 DNA binding 3.86E-02 (1.2)
ynTTATCC	6.75	SREATMSD; AT_I-box	**TTATCC**; **CCTTATCC**T	
nGTTGACw (d)	5.31	ZM_O2-site	**GTTGAC**GTGA	GO:0006952 defense response 2.99E-04 (1.9); GO:0009607 response to biotic stimulus 3.56E-04 (1.7); GO:0016301 kinase activity 7.52E-11 (1.7)
TTTGCnrA	6.13			GO:0016773 phosphotransferase activity, alcohol group as acceptor 1.14E-02 (1.6); GO:0016772 transferase activity, transferring phosphorus-containing groups 2.60E-02 (1.5)
rATyTGGG	5.58			
TrTwTATA	9.35	AT_TATA-box	**TATATA**A	GO:0019748 secondary metabolism 2.76E-02 (2.1); GO:0006519 amino acid and derivative metabolism 1.35E-02 (1.8); GO:0003700 transcription factor activity 3.36E-02 (1.3)
ATArwACA (e)	5.79	OS_Unnamed_2	CCA**TGTCATAT**T	
nTTCCCGC (5)	27.27	NT_E2Fa	**TTTCCCGC**	GO:0006261 DNA-dependent DNA replication 6.48E-04 (6.2); GO:0000067 DNA replication and chromosome cycle 1.06E-07 (5.5); GO:0006260 DNA replication 3.57E-05 (5.1)
TkAGAwnA	8.86	BO_TCA-element3	**TCAGAAGA**GG	GO:0006464 protein modification 4.52E-02 (1.7); GO:0003824 catalytic activity 5.20E-03 (1.1)
AAACCCTA (13) (f)	40.06	TELOBOXATEEF1AA1	**AAACCCTA**A	Ribosome biogenesis and assembly 9.86E-13 (4.4); ribosome biogenesis 5.67E-12 (4.3); pre-mRNA splicing factor activity 3.20E-04 (3.9)
mGnyAAAG (g)	6.38			GO:0003824 catalytic activity 2.93E-02 (1.1)
GAnCnkmG	6.29			GO:0003729 mRNA binding 1.00E-02 (3.1); GO:0003735 structural constituent of ribosome 3.69E-02 (1.7); GO:0006412 protein biosynthesis 3.15E-03 (1.7)
TCnCTCTC	8.98	LE_5UTRPy-richstretch	TT**TCTCTCT**CTCTC	GO:0003777 microtubule motor activity 9.90E-03 (2.7); GO:0050789 regulation of biological process 2.27E-03 (1.4); GO:0016772 transferase activity, transferring phosphorus-containing groups 7.89E-03 (1.4)
wmGTCmAm	7.16			GO:0003824 catalytic activity 4.51E-03 (1.1)
ynCAACGG	8.39	CR_MSA-like	***YCYAACGG*** *YYA*	GO:0003777 microtubule motor activity 3.17E-03 (3.4); GO:0003774 motor activity 8.55E-03 (2.9)
nmGATyCr	5.66			GO:0006944 membrane fusion 2.32E-02 (4.5); GO:0003735 structural constituent of ribosome 2.77E-03 (1.9); GO:0005198 structural molecule activity 7.11E-04 (1.9)
CGkCGmCn	7.68	OS_GC-motif5	**CGGCGCCC**T	
AGGCCCAw (9)	21.94	UP1ATMSD	**GGCCCAW**WW	GO:0007046 ribosome biogenesis 3.56E-14 (4.3); GO:0042254 ribosome biogenesis and assembly 2.28E-14 (4.3); GO:0003735 structural constituent of ribosome 8.66E-29 (3.3)
AykyATwA	6.09			
CTGnCTCy	6.91			GO:0016301 kinase activity 3.44E-02 (1.3); GO:0003676 nucleic acid binding 3.48E-02 (1.2); GO:0005488 binding 2.60E-03 (1.2)
TsTCGnTT	7.22			GO:0003824 catalytic activity 5.10E-03 (1.1)
TmAsTGAn	7.76	OS_GTCAdirectrepeat	TAAGTCA**TAACTGATG**A	GO:0016491 oxidoreductase activity 3.85E-03 (1.5); GO:0008152 metabolism 5.74E-03 (1.2); GO:0003824 catalytic activity 5.70E-04 (1.2)
yyACrCGT (2)	6.56	ST_G-box	**TCACACGT**GGC	GO:0009605 response to external stimulus 4.80E-02 (1.6); GO:0006950 response to stress 3.42E-02 (1.6)
mATATTTT	5.51	GM_Nodule-site1	GATATATT**AATATTTT**ATTTTATA	
CCAATnCm	5.78	CAATBOX1; HV_ATC-motif	**CAAT; **G**CCAATCC**	GO:0008152 metabolism 2.01E-02 (1.2)
rkTCAwGm	5.42			GO:0003824 catalytic activity 6.17E-05 (1.2)
ssCGCCnA (2)	9.13	E2F1OSPCNA	**GCGGG**AAA	GO:0000067 DNA replication and chromosome cycle 4.74E-02 (3.0); GO:0006259 DNA metabolism 2.15E-03 (2.3); GO:0007049 cell cycle 4.29E-02 (2.2)
TTTATGnG	7.1			
TCAwATAA	6.74			

*Numbers in parentheses indicate the number of clusters (containing co-expressed genes) in which the motif was independently identified. The letters in parenthesis refer to the updated TFBS identified using the two-way clustering: (a) GCAAnTCn; (b) GTACmwGy; (c) yCATTTAT; (d) mkTTGACT; (e) ATrrwACA; (f) AAACCCTA; (g) mGnCAAAG. ^†^Network-level Conservation score. ^‡^Residues in bold indicate the matching position between the known motif and the motif found in this study. Known motifs were retrieved from PLACE [26] and PlantCARE [27]. ^§^Only the first three GO categories according to the highest enrichment score are shown. The enrichment score is shown as number in parentheses.
